# Congenital radial head dislocation with a progressive cubitus valgus: a case report

**DOI:** 10.1007/s11751-011-0126-z

**Published:** 2012-01-06

**Authors:** Laurens Kaas, Peter A. A. Struijs

**Affiliations:** 1 Department of Orthopaedic Surgery, Academic Medical Center, Amsterdam, The Netherlands; 2 Bethaniëplein 10, 3701 EM Zeist, The Netherlands

**Keywords:** Congenital radial head dislocation, Cubitus valgus, Elbow, Anomaly, Treatment, Review

## Abstract

Congenital dislocation of the radial head is rare, although it is the most common congenital anomaly of the elbow. A concomitant progressive cubitus valgus of the elbow has not previously been described in literature. We describe a case of an 8-year-old girl with an unilateral congenital radial head dislocation with a progressive cubitus valgus of 35°, caused by a prematurely closing physis of the lateral humeral condyle. This might be caused by an increased pressure on the lateral physis by the anteriorly dislocated radial head. As no complaints or limitations were present, treatment was non-operative with clinical observation, with satisfactory results after a follow-up of 18 months. A concomitant progressive cubitus valgus can be present in patients with a congenital radial head dislocation. Non-operative treatment can provide satisfactory results.

## Introduction

Chronic radial head dislocation can be either congenital, developmental or post-traumatic [1, 2]. Congenital dislocation of the radial head is rare, although it is the most common congenital anomaly of the elbow. It can occur uni- and bilateral and has been described as an isolated abnormality, as well as a feature of a number of syndromes [3–5]. The diagnosis is often not made at birth, but when children present with limitation in range of motion (ROM), deformity of the elbow or elbow pain [2, 3]. Dislocation is usually towards posterior (65%) [2]. We present a case of an 8-year-old girl with unilateral congenital anterior radial head dislocation with a progressive cubitus valgus caused by a premature physeal closure or a growth arrest of the lateral humeral condyle. In girls, this growth plate normally closes at about the age of 12–8 years, with a range from 9–6 to 15–0 years. The physis of the medial epicondyle of girls closes at the age of 11–0 to 16–0 years, with a mean of 14–1 years [6]. Additionally, we review the literature on congenital dislocation of the radial head.

## Case report

**Fig. 1:**
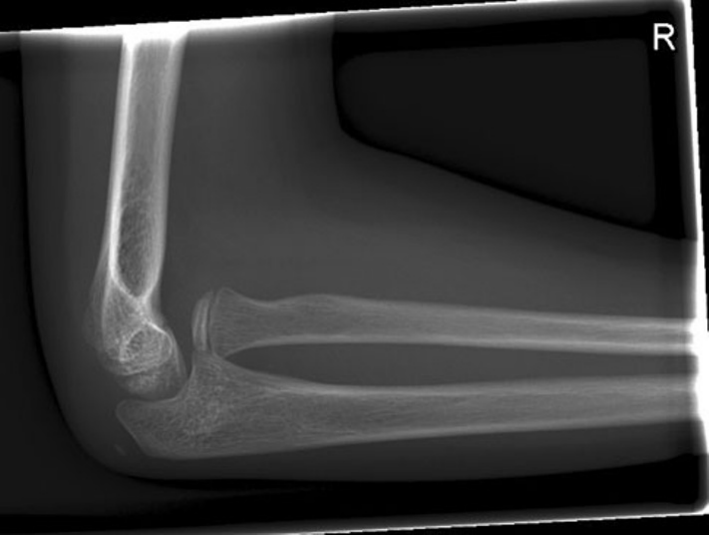
Lateral radiograph of the patients’ right elbow. Note the dislocation of the radial head

**Fig. 2:**
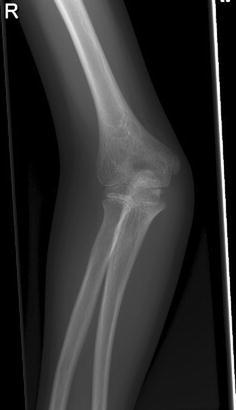
Anteroposterior (AP) radiograph of the elbow. Note the valgus angle and the dislocation of the radial head

**Fig. 3:**
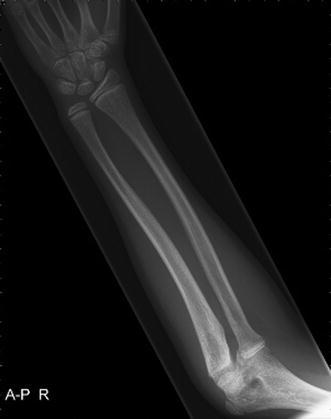
AP radiograph of the right forearm. A mild bowing of the ulna can be observed

**Fig. 4:**
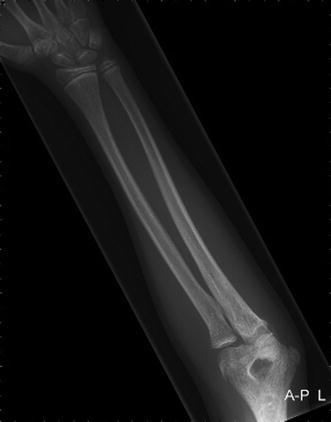
AP radiograph of the unaffected left forearm

**Fig. 5:**
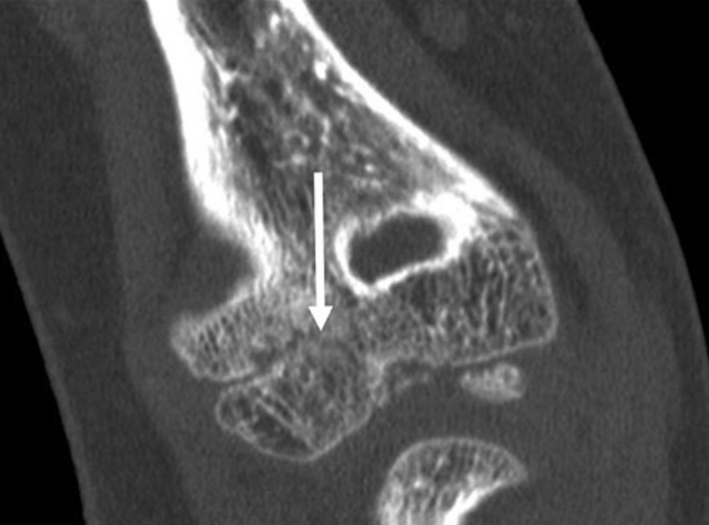
Sagital view of the CT-scan of the patients’ right elbow. The partially ossifying physis with boney bridges of the lateral humeral condyle is marked with an *arrow*

An 8-year-old girl with a rapidly increasing valgus angle of the right elbow presented at the out-patient clinic of our hospital. No evident trauma of the affected elbow had occurred. In another hospital, she had been diagnosed with a unilateral congenital radial head dislocation of the right elbow at the age of 3 months, for which a conservative treatment was initiated. Besides the increasing deformity, the patient had no complaints of pain or limitations. On physical examination, the patient was found to have a full and painless ROM and a normal carrying angle of the left elbow. The right elbow showed a cubitus valgus with clinically a carrying angle of 32°. The dislocated radial head was not painful on palpation. The range of motion was nearly full: flexion 130°, extension 10°, pronation 75° and supination 80°, compared with a flexion of 140°, extension of 0°, pronation of 80° and supination of 80° of the contralateral elbow. Plain radiographic evaluation of the right elbow showed an anterior dislocation and deformation of the radial head, with a long narrow neck and a hypoplastic capitellum and an increased valgus angle (Figs. 1 and 2). The left elbow showed no abnormalities. Radiographs of both forearms show a mild ulnar bowing on the right side, as compared to the left side (17 and 12°) (Figs. 3 and 4). This is probably due to chronic dislocation of the radial head. CT imaging of the elbow showed the anterior dislocation of the radial head with an overgrowth of the radius of 8 mm and a normal ulnohumeral joint. There is a premature ossifying physis of the lateral humeral condyle (Fig. 5). Whilst no complaints or limitations were present, treatment was non-operative with clinical observation. No restrictions in daily elbow use were advised. The result of this treatment strategy was satisfactory after a follow-up of 18 months. No increase in valgus angle was seen, and elbow function was not painful. Treatment of the valgus angle can be observational. In case of progression, a supracondylar varus osteotomy can be considered.

## Discussion

To our best knowledge, the combination of congenital radial head dislocation and cubitus valgus has never been described in literature. Premature closure of the physis of the lateral condyle seems the cause on CT imaging. This growth arrest might have several causes, for example, trauma, infection, irradiation or bone tumours [7–9]. An altered physical pressure on the open physis can result in a stimulatory or inhibitory effect on growth and extreme forces can even fully inhibit growth. However, much milder forces can inhibit or modify the epiphyseal growth rate [6]. The capitellum generally becomes visible in the first or second year of life. During maturation, the capitellum fuses with the trochlea and the lateral epicondyle, before it unites with the humeral shaft at a median age of 12–8 years in girls, with a range from 9–6 to 15–0 years [6]. In our case, anterior dislocation and overgrowth of the proximal radius might have resulted in an increased pressure or repetitive minor trauma on the lateral physis, causing the growth arrest with an additional cubitus valgus, as a result of continuing growth of the medial epiphysis. An unnoticed trauma could also be the cause of the physeal growth arrest [6].

The effects of cubitus valgus can be function loss and cosmetic disturbance [10, 11]. The goal of corrective surgery is restoration of alignment and range of motion. Indications for surgical intervention are similar to surgical correction of a cubitus varus deformity: functional limitation, elbow pain and cosmetic reasons. Several osteotomy techniques are described, such as closing wedge osteotomy, corrective dome osteotomy and step-cut osteotomy. Functional outcomes are generally good. Elbow stiffness, ulnar nerve injury and persistent deformities are the most commonly reported complications [12].

The exact cause of congenital radial head dislocation remains unknown. There have been reports of familial occurrence, but no definitive hereditary pattern has been established [3, 13–15]. Some believe that an abnormal capitellum formation in utero lays at the base of the disorder [2, 3, 5]. (Unrecognized) Trauma had also been stated to be the cause of radial head dislocations in newborn, especially if it occurs unilateral. A pulled elbow of infancy resulting in radial head (sub) luxation and laxity of the annular ligament may occur as shortly after birth. If not treated by closed reduction, persistent radial head dislocation may result in similar deformities as in children with a congenital radial head dislocation [1, 3]. Developmental causes of radial head dislocation include an inadequate length of the radius and multiple hereditary osteochondromatosis [1].

A wide variety of reported clinical morphology of the proximal radius, ulna and distal humerus exists. The annular ligament can be normal [16], dysplastic [17] or absent [18]. Posterior dislocation is most common (65%). Anterior dislocation occurs in 18% of the cases and lateral in 17% [2]. Radiographic diagnostic criteria as described by McFarland are (1) relative shortening of the ulna or overlength of the radius, (2) absence or hypoplasia of the capitellum, (3) grooving of the distal radius, (4) prominent ulnar epicondyle, (5) a partially defective trochlea and (6) a dome-shaped radial head with a long narrow neck [19]. Additional criteria that favour a congenital dislocation are described by Mardam-Bey and Ger: (1) bilateral involvement and concurrence of other congenital anomalies, (2) familial occurrence, (3) dislocation seen at birth and (4) no history of trauma [2]. Ulnar bowing has also been described as an additional criterium [16, 17].

As the symptoms in childhood are usually mild, observation is the standard treatment. However, occasional pain and functional limitations may be experienced [5, 13, 20]. Painful elbow snapping as a result of annular ligament interposition during flexion and extension also has been reported [21]. Some children develop increasing elbow pain during adolescence [16]. Surgery can be indicated because of pain, functional impairment, snapping or cosmetic reasons. Various surgical treatment possibilities have been discussed in literature, for example, resection, rotation osteotomy [13], ulnar osteotomy and reconstruction of the annular ligament [18]. Resection is advised in a symptomatic patient after reaching skeletal maturity, because of the risk of regrowth if performed too early. Excision usually relieves pain, but does not always lead to significant functional improvement. Complications include pain in the distal radio-ulnar joint due to proximalization of the radius, instability, valgus deformation, weakness and regrowth of the proximal radius [2, 3, 5, 20, 22]. Open reduction, in combination with osteotomy of radius and/or ulna in younger patients is reported to be successful [17, 18, 23, 24]. To prevent redislocation in radial head sparing surgery, reconstruction of the annular ligament can be performed [16–18]. The results of surgical treatment of congenital radial head dislocations in literature since 1979 are summarized in Table 1. These publications consist of retrospective case reports or case series with a small numbers and heterogenous patient groups. No randomized comparison between surgical and conservative treatment has been made.

**Table 1:** Summary of surgical results in studies performed since 1979

First author	Year	Number of patients/elbows	Age (range)	Post-traumatic/congenital	Direction of dislocation	Treatment (number of patients)	After treatment	Follow-up (years)	Results
Mardam-Bey [2]	1979	50/77	6 (0–18)	0/77	A: 14 P: 50 L: 13	Conservative (70) Excision (7)	n.d.	1.3–39	Conservative: occasional pain, functional limitation, cosmetic significant radial head prominence Excision: less pain, but no functional improvement
Kelly [3]	1981	8/12	0–33	0/12	A: 3 P: 8 Unknown: 1	Conservative (4) Excision (8)	n.d.	n.d.	Conservative: n.d Excision: no improvement in ROM, but satisfied patients 2 patients with regrowth
Lancaster [25]	1985	1/1	23	0/1	LS: 1	Excision (1)	n.d.	0.5	Increased pro/supination
Miura [5]	1990	34/45	0–30+	0/34	A: 24 P: 20 L: 1	Conservative (31) Rotation osteotomy + ulna osteotomy (2) Excision (1)	n.d.	n.d.	Conservative: n.d Surgery: 2 successful, 1 unsuccessful
Bell [20]	1991	18/20	7.8 (5–18)	0/20	P: 16 PS: 4	Excision (12) Conservative (8)	n.d.	7.8 (2.5–13.5)	Conservative: increased pain in 1 Excision: 5 no pain, 2 less pain, 5 no effect. No consistent improvement in ROM
Campbell [22]	1992	6/8	13 (10–15.5)	0/8	P: 5 PL: 3	Excision (8)	n.d.	7 (2–19)	Good: 5 Fair: 2 Poor: 1, due to regrowth: re-excision
Futami [24]	1992	5/6	13 (8–19)	1/5	A: 6	Rotation osteotomy (6) Radial shortening (2)	External fixation 3 weeks	7 (3–10)	Improved ROM of 10° Limited rotation in 1
Sachar [16]	1998	10/12	2 (0.5–6)	0/12	A: 3 P: 8	Resuturing annular ligament (12)	K-wire 6–8 weeks	1.8 (0.3–3.4)	Redislocation in 2. Improvement ROM
Kim [23]	2002	14/15	9.5 (3–15)	12/3	A: 13 AL: 1 P: 1	Annular ligament reconstruction (triceps autograft) (15) Radial shortening (7) Rotational osteotomy (3) Radial head arthroplasty (2) Ulnar flexion osteotomy (9) PRUJ notchplasty (2)	K-wire (6) or plaster (9) 3 weeks	3.6 (0.3–15)	Excellent: 10 Good: 2 Fair: 2 Poor: 1
Yamazaki [18]	2007	1/2	5	0/2	AL 2	Reconstruction annular ligament + ulna osteotomy (m. extensor carpi ulnaris fascia autograft) (2)	Plaster 6 weeks	9.2	Nearly full ROM, no pain or limited function in daily activities

*A,* anterior; *P*, posterior; *L*, lateral; *AL/PL*, antero/posterolateral; *LS/PS*, lateral/posterior subluxation; *PRUJ*, proximal radio-ulnar joint, *n.d.,* not described, *ROM,* range of motion

## Conclusion

Congenital dislocation of the radial head is rare, although it is the most common congenital anomaly of the elbow. A concomitant progressive cubitus valgus can be present in patients with a congenital radial head dislocation. This might be caused by an increased pressure on the lateral physis by the anteriorly dislocated radial head or an unnoticed trauma. If no complaints or limitations are present, non-operative with clinical observation can lead to satisfactory results.
